# Epinephrine-Containing Digital Nerve Block: A Case of Digital Tip Necrosis Leading to Amputation in a Patient With No Known Vascular, Rheumatologic, or Smoking History

**DOI:** 10.1016/j.jhsg.2021.03.005

**Published:** 2021-04-27

**Authors:** Ian S. Hong, Nancy J. Moontasri, David F. Ratliff

**Affiliations:** ∗ New York Medical College, School of Medicine, Valhalla, NY; † Department of Orthopedics, St. Joseph’s Regional Medical Center, Paterson, NJ

**Keywords:** Amputation, Epinephrine, Fingertip, Local anesthetic, Necrosis

## Abstract

The use of epinephrine-containing digital nerve blocks has been shown to be safe in recent literature, challenging the historical fear of complications arising from irreversible ischemia. We present a rare case of digital tip necrosis following the injection of lidocaine-containing epinephrine for the purpose of wart removal using cryotherapy, ultimately requiring amputation.

The earliest reported case of digital necrosis following local anesthetic (LA) injection dates back to 1931.^1^ The investigator reported 4 cases of digital gangrene after injecting an unreported volume of 0.5% to 1% novocaine, in combination with a tourniquet, to induce a digital block.^1^ The tourniquet functionally produced similar effects as epinephrine—a common adjuvant therapy for LA—by decreasing local blood flow and prolonging the duration of anesthesia. Historically, the use of epinephrine was thought to cause irreversible arterial insufficiency, leading to distal digital tip necrosis. However, numerous studies have shown its safety.^2^, ^3^, ^4^ Although recent literature supports its relative safety, we present a case of digital tip necrosis leading to amputation in a healthy patient following the administration of an epinephrine-containing digital nerve block.

## Case Presentation

A 57-year-old woman with a past medical history of hyperlipidemia and no known vascular, rheumatologic, or smoking history was referred to hand surgery department for the swelling of her right small finger. She was employed as a packaging specialist and denied any previous trauma or ipsilateral hand injuries. The day before, she had received a 3-mL digital block of 1% lidocaine with 1:100,000 epinephrine by her dermatologist for the removal of 4 warts. She reported that the block was placed at the volar proximal phalanx. There was no tourniquet used. Shortly after, the patient noticed swelling and blistering that progressed throughout the day. Dressings were reportedly not tight. She proceeded with warm water soaks. She and her family reported that the water was checked and was warm but never scalding. The patient then returned to her dermatologist the following day for worsening symptoms. The dermatologist drained a large blister on the finger, and she was referred for orthopedic evaluation.

**Figure 1:**
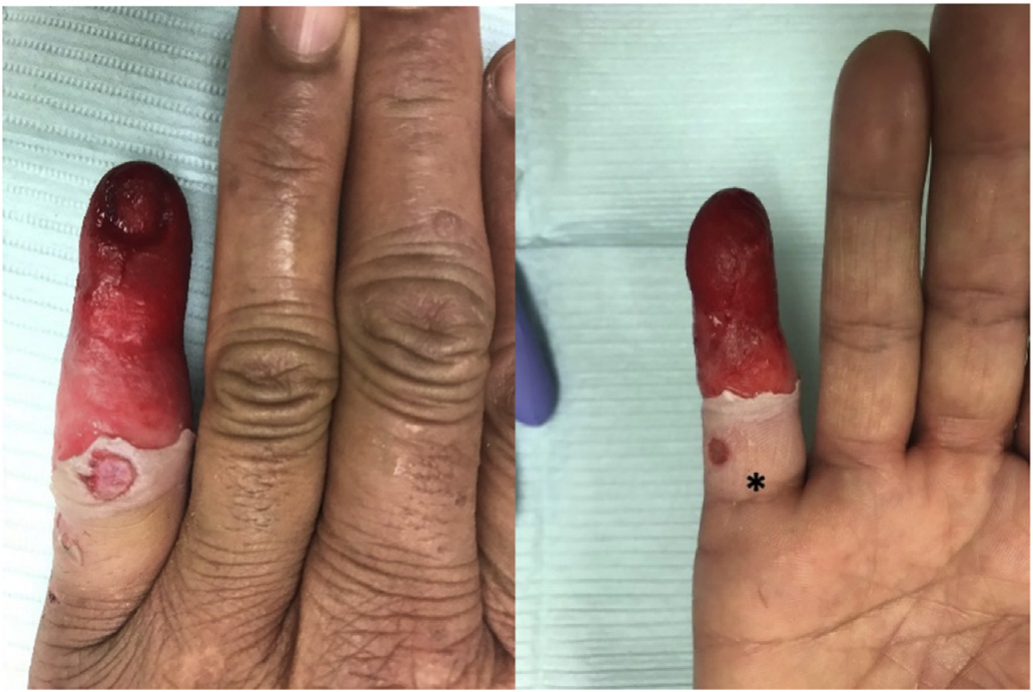
Patient’s finger at 28 hours after injection. The patient received a 3-mL digital block of 1% lidocaine with 1:100,000 epinephrine by her dermatologist on the volar aspect of the proximal phalanx (denoted by ∗).

On examination, there was superficial skin separation with serosanguinous fluid from the proximal interphalangeal joint distally, which presented as a large blister encompassing the entire finger distally. After it was unroofed, there was superficial epidermal loss, but the exposed area appeared viable, with oozing bleeding (Fig. 1). The nail was loose and came off with the blister. The small fingertip was cooler to touch, and there was decreased light touch sensation, with pressure sensation intact. There was no evidence of purulence, and the finger was soft throughout. She was treated with local wound care.

**Figure 2:**
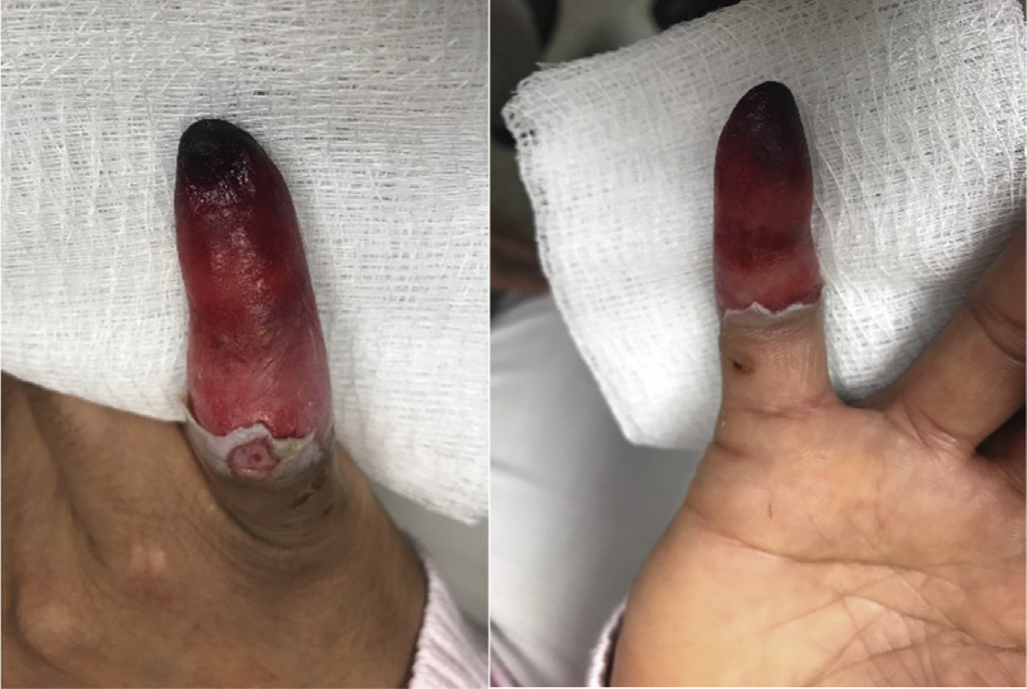
Patient’s finger on day 11 after injection.

Over the next several weeks, the patient was monitored closely. She continued with local wound care. She was prescribed aspirin and nitropaste early to try to maximize the blood flow to the finger. She stopped using nitropaste, as she felt that it worsened the swelling. She had diffuse dorsal proximal interphalangeal joint swelling with superficial watery drainage. The distal fingertip became darker and lost all sensation by day 12 after the injection. Distal superficial tip necrosis was 7 mm of the tip on day 12 (Fig. 2) and was at 17 mm of necrosis with the loss of turgor by day 19. A computed tomography angiogram demonstrated no flow distal to the proximal interphalangeal joint of the small finger but was otherwise normal. The patient was counseled to allow the necrosis to demarcate.

**Figure 3:**
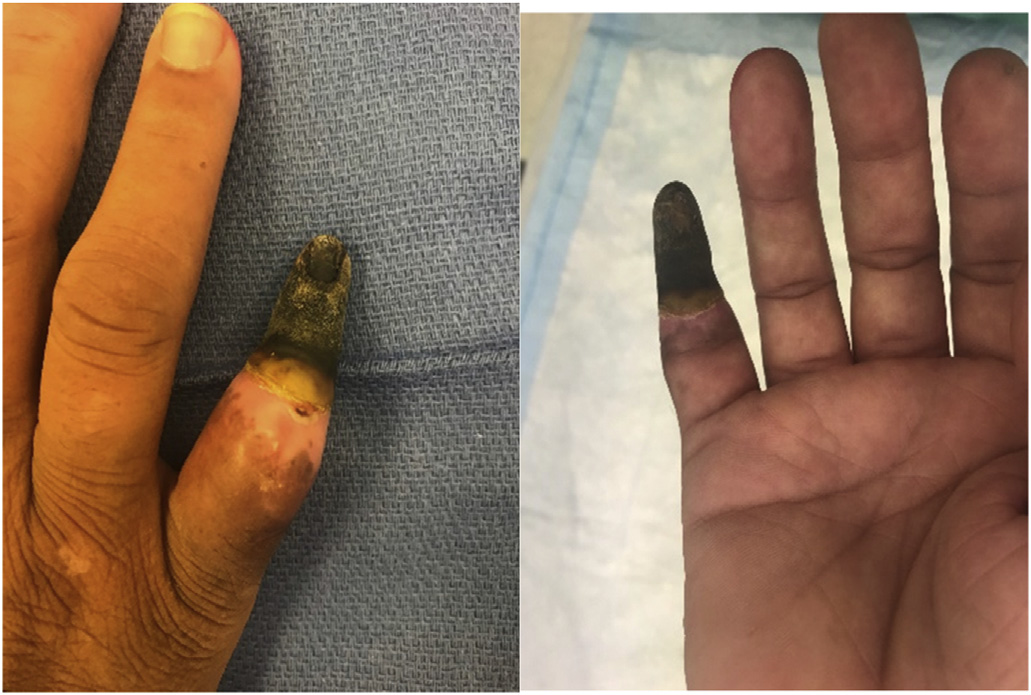
Patient’s finger on day 59 after injection.

Approximately 1 month after the initial evaluation, there was necrosis distal to the midportion of the middle phalanx, with complete firm dry gangrene and with no supple soft tissue palpable beneath. At 8 weeks after the initial presentation (Fig. 3), the patient opted for a right small finger amputation. The amputation was performed at the midportion of the middle phalanx of the right small finger, with local coverage. She did well with hand therapy and was able to return to work 9 weeks after surgery; she was discharged at 13 weeks after surgery, with follow-up at her discretion.

## Discussion

In their 1944 *Surgery of the Hand* textbook, Bunnell and Boyle^5^ state that epinephrine-containing LA should be avoided in the digits because gangrene often results. In 2007, Thomson et al^6^ conducted an in-depth literature review of digital necrosis cases involving local anesthesia and found 48 cases from 1880 to 2005. Of the 48 cases, 42 occurred prior to the 1950s, during an era where procaine was the predominant form of LA and expiration date labels were not required for injectables. Procaine was later found to undergo spontaneous hydrolysis during storage, increasing its acidity to levels known to cause tissue necrosis.^6^ Of note, the authors did not report any cases of digital infarction from lidocaine.

Currently, there are no reports of irreversible digital necrosis following accidental digital injection with epinephrine autoinjectors, which are used for the treatment of anaphylaxis and allergic reactions. In these cases, patients reported pain with paresthesia and, on examination, the digit was cold and blanched, with poor capillary refill times.^4^ Muck et al^4^ sought to investigate the effects on digits of accidental deployment of epinephrine autoinjectors. In their retrospective review of 6 poison centers over a period of 6 years, all patients had the resolution of their symptoms, with no need for any surgical care. The conclusion was that the concentrations of epinephrine found in these autoinjectors far exceeded the concentrations found in commercially available LAs, supporting the use of epinephrine in digital injections.

Finger compartment syndrome is a rarely mentioned entity. Although the finger is not a standard fascial compartment, dorsal and volar “compartments” of the finger are separated by Grayson and Cleland ligaments, which contain the digital neurovascular structures. Increased compartment pressures or a decrease in the size of the compartment can be caused by a wide array of etiologies, including injections of LA. In a retrospective review, an average volume of 4.3 mL, ranging from 0.5–10 mL, of 1% lidocaine with epinephrine (1:100,000) was used for digital nerve block without any complications. Although there is no current literature that elucidates a safe volume that the hand compartments can tolerate, the range of volume injected in the aforementioned study show that 10 mL of LA with epinephrine can be administered within the hand without inducing compartment syndrome.^3^ However, there is no standardized digital block technique, and techniques/locations vary. Authors have recommended using caution with a circumferential ring of fluid due to pressure.^7^ More research would have to be done regarding the location and volume of fluid, but digital necrosis is such a rare injury, and further research is difficult to produce.

A previous 2014 case report by Ruiter et al^8^ describes a 16-year-old woman with no known vascular, rheumatologic, or smoking history requiring finger amputation subsequent to LA injection with epinephrine for wart removal. The authors do not mention the concentration and volume of LA with epinephrine injected or the specific treatment protocol used prior to irreversible digital necrosis, limiting comparative value with our case report. However, similar to our case, phentolamine, which has been shown to reverse the adverse effects of epinephrine, was not used as a part of the medical management.^2^^,^^6^^,^^9^ The vasospasm of epinephrine would be expected to be resolved with phentolamine, which is reported to be effective when given up to 13 hours after the initial injection.^9^

Cryotherapy is considered a safe method of wart removal, with few severe side effects, and its mechanism of action involves vascular stasis and occlusion.^10^ We propose the possibility of a synergistic adverse effect of epinephrine and cryotherapy, causing blistering of the digits and inflammation beyond the intended therapeutic effects. Two case reports of digital necrosis following injection of LA with epinephrine certainly do not amount to causation, but many questions remain unanswered. As noted above, contributing factors could be local pressure phenomena (ie, compartment syndrome) because of the location of the block or a possible ring-type block. In this case, the patient had reported that her dressings were not tight and there was no hot water used, either of which could have been a potential contributing cause.

The preponderance of evidence is that epinephrine is overwhelmingly safe. Although literature has shown that epinephrine is generally well tolerated in the digits, further research may be required to avoid the rare complications found in this case report. This case is reported to add to the body of literature regarding complications following digital block, with the true etiology of complications unknown. Although most effects of epinephrine in the digits may be transient, we present a case where, following the injection of low-concentration epinephrine in a healthy individual for cryotherapy, the patient had digital necrosis ultimately requiring an amputation.
